# Association between the school physical activity environment, measured and self-reported student physical activity and active transport behaviours in Victoria, Australia

**DOI:** 10.1186/s12966-021-01151-6

**Published:** 2021-06-22

**Authors:** Nicholas Crooks, Laura Alston, Melanie Nichols, Kristy A. Bolton, Steven Allender, Penny Fraser, Ha Le, Joanne Bliss, Claire Rennie, Liliana Orellana, Claudia Strugnell

**Affiliations:** 1 Global Obesity Centre (GLOBE), Institute for Health Transformation, Faculty of Health, Deakin University, 1 Gheringhap Street, Geelong, Victoria 3220 Australia; 2 Deakin Health Economics, Institute for Health Transformation, Faculty of Health, Deakin University, 221 Burwood Hwy, Burwood, Geelong, Victoria 3125 Australia; 3 Biostatistics Unit, Faculty of Health, Deakin University, 221 Burwood Hwy, Burwood, Geelong, Victoria 3125 Australia

**Keywords:** Physical activity, School environments, School policies, Children, Active transport

## Abstract

**Background:**

Environments within schools including the physical, social-cultural and policy/practice environments have the potential to influence children’s physical activity (PA) behaviours and weight status. This Australian first study comprehensively examined the association(s) of physical, social-cultural and policy/practice environments with PA, active transport (AT) and weight status among regional primary school children.

**Methods:**

Data were from two childhood obesity monitoring systems in regional Victoria, Australia. Measured height and weight were collected from students in Year 2 (aged approx. 7–8 years), Year 4 (9–10 years), and Year 6 (11–12 years). Self–reported PA behaviour, including AT were collected from students in Year 4 and 6 and a sub-sample wore an ActiGraph (wGT3X-BT) accelerometer for 7-days. A school physical activity environment audit was completed by the school principal and responses were used to calculate school physical activity environment scores (PAES) and active transport environment scores (ATES). Mixed effects logistic regression was used to assess the relationship between the proportion of students meeting the PA guidelines (≥60mins/day of moderate-to-vigorous PA) and PAES tertiles (low, medium, high) and those using AT and school ATES tertiles, controlling for gender, school size/type and socioeconomic composition.

**Results:**

The analysed sample included 54/146 (37%) schools and 3360/5376 (64%) students. In stratified analysis, girls in schools with a medium PAES score were more likely to meet the objectively measured PA guideline compared to low PAES score (OR 2.3, 95%CI 1.27, 4.16). Similarly, students in schools with a medium or high ATES score had higher odds of self-reported AT (medium OR 3.15, 95%CI 1.67, 5.94; high OR 3.71, 95%CI: 1.80, 7.64). No association between PAES or ATES and weight status were observed. Self-reported AT among boys (OR 1.59, 95%CI 1.19, 2.13) and girls (OR 1.56, 95%CI 1.08, 2.27) was associated with higher odds of meeting self-reported PA guidelines on all 7-days than those who did not report using AT.

**Conclusions:**

In this study of regional Victorian primary schools, PA environments were only associated with girls’ adherence to PA guidelines. School AT environments were strongly associated with students’ AT behaviours and with increased likelihood of students being physically active.

**Supplementary Information:**

The online version contains supplementary material available at 10.1186/s12966-021-01151-6.

## Background

One quarter of Australian children, aged 5–17 are with overweight or obesity [1]. Increased weight status in childhood strongly persists into adulthood [2], increasing risks of adverse physical, mental and social health outcomes, including the development of non-communicable diseases, reduced life-expectancy and poor mental health [3]. Inadequate physical activity and excessive sedentary time among young people remain key challenges globally [4] and have contributed to increased prevalence of overweight and obesity among children [5].

Given the large proportion of time children spend at school during their formative years, schools are a key setting in which to promote healthy habits including being physically active [6]. There is also evidence that engaging in adequate levels of physical activity (PA) has benefits for students’ classroom behaviours and academic performance [7]. The World Health Organization’s (WHO) Health Promoting Schools framework identifies key areas to promote PA in schools; 1) formal health curriculum; 2) ethos and environment of the school; and, 3) engagement with families and/or communities [6]. PA promotion is operationalised formally in schools via physical education (PE) and sport education classes in Australia. The state of Victoria mandates that all students in the first four years of primary (elementary) school (Prep to Year 3) receive 20 to 30 min of PE each day and that all students in Years 4 to 6 receive three hours per week of PE and sport education (SE), with a minimum of 50% of time devoted to PE [8]. While officially mandated, there are no formal or informal consequences if schools do not achieve these requirements.

Barriers to PE provision in schools include time constraints within a crowded curriculum, and a lack of specifically trained PE teaching staff [9]. Additionally, PE teachers report a range of student-level barriers such as difficulty in engaging students, a lack of student interest in PE [10], as well as limited basic skill competencies [11] and decreased interest in traditional forms of structured PA such as team sports [12]. Given these limitations, there is a need to look more holistically at how the school and its environment can promote large-scale and sustainable improvements in PA levels [6].

Schools’ physical, policy and practice environments are crucial in increasing opportunities for children to be active [6]. Sufficient space and facilities for students to be physically active as well as the provision of both fixed and mobile equipment have the potential to increase students’ PA during recess and lunch breaks [13]. Supportive environments can also play a part in setting the culture within the school around PA, including staff role modelling PA as well as engaging parents/guardians in sports days and active transport initiatives.

Utilising active transport (AT) modes (predominantly walking, cycling, and/or scooting) to or from school have been shown to increase the total amount of PA accrued by students over the day, and contribute to meeting PA guidelines [14]. Initiatives to promote AT include Walking School Bus style programs for walking or cycling to school, as well as the promotion of safe routes to school [15], and infrastructure or physical environment supports such as the presence of supervised crossing outside the school, traffic calming measures, student drop-off zones and secure bike parking [16].

Despite the wide range of potential school environment policies and initiatives targeted at increasing physical activity for students, there is limited evidence on how the policy and physical characteristics of the school environment correlate with students’ PA behaviours and weight status. This study aims to understand associations of policy, structural and cultural elements within the school environment with the proportions of students meeting PA recommendations, using AT to and from school, and classified as having overweight or obesity.

### Aims

1. To understand associations between characteristics of the school physical, social-cultural and policy/practice environments with students’ physical activity and active transport use levels, and;
2. To assess the associations between the school physical activity and active transport environments and odds of having overweight and obesity within a school.

It is hypothesised that children within schools with greater physical, sociocultural and policy/practices supporting PA and AT will have higher rates of PA and AT and lower rates of overweight and obesity.

## Methods

### Sampling

Data were collected from two large community-based childhood obesity and risk factor surveillance systems across nine Local Government Areas covering 36,091 km^2^ of regional Victoria, Australia. Data came from the Great South Coast Childhood Obesity Monitoring study located in South-West Victoria [17] and the Goulburn Valley Health Behaviours Monitoring study in North-Eastern Victoria, conducted in 2017 and 2016 respectively. Both studies employed the same sampling and data collection methods described previously [17]. In brief, all primary schools (Government, Independent and Catholic) in both study regions were invited to participate via letter to the principal. An initial visit to each school was typically conducted to confirm school participation and/or to distribute the plain language statements and recruitment forms and explain the study to students. All students in Year 2 (aged approx. 7–8 years), Year 4 (aged approx. 9–10 years) and Year 6 (aged approx. 11–12 years) at participating schools were invited to take part. Both studies used an opt-out recruitment process whereby students who did not want to participate returned a signed form by their parent/guardian to decline participation or verbally declined to participate on the day of testing. The trained staff visited each school to conduct the anthropometric measures (height and weight) with all students and behavioural surveys with Year 4 and Year 6 students and the school environment audit with the school principal.

#### Measures

##### Child measurements

Height and weight were measured by trained staff in a private booth. Height was measured to the nearest 0.1 cm and weight the nearest 0.05 kg. All students were measured twice and where the two initial measures differed by more than 0.5 cm and 0.1 kg for height and weight respectively a third measurement was taken. The mean of all measurements was used in analyses.

Students completed an electronic self-reported questionnaire (see Supplementary File 1) individually on electronic tablets, with support when needed from trained staff, which took approximately 30–45 min. This paper reports on data collected in two sections of the questionnaire.

1. Demographic information; name, date-of-birth, gender, postcode, country of birth, ancestry, Aboriginal and or Torres Strait Islander status and language spoken at home.
2. Physical activity, sedentary behaviour and active transport were recalled using modified questionnaire items from the Core Indicators and Measures of Youth Health survey [18] and School Health Action, Planning and Evaluation System (SHAPES) questionnaire [19]. Daily amount of moderate-to-vigorous physical activity both within and outside the school day, time spent engaged in screen-based activities, outside of school work, such as watching television, gaming and using social media, active transport use to/from school, parental support and encouragement for physical activity.

A randomly selected sub-sample of Year 4 and Year 6 students at each school were invited to wear a waist-worn accelerometer (ActiGraph wGT3X-BT, ActiGraph LCC, Pensacola, US) for the next 7 days. Students were asked to wear the device at all times except for sleeping, bathing and when involved in contact or water sports.

##### School level measurements

A school environment audit (Supplementary File 2) was completed by the school principal on the day of student measurements. This tool was adapted from the Be Active Eat Well school environment audit [20] and The International Study of Childhood Obesity, Lifestyle and the Environment (ISCOLE) school environment audit tool [21]. Questions inquired about the policies and practices around the promotion of PA within the school and the perceptions of policy effectiveness. Questions around the provision of the mandated amount of PE and Sport Education (SE), whether the school employed a qualified PE teacher and questions about support from parents/guardians and role-modelling from teachers around PA were also asked. Policies and practices concerning the promotion and support for the use of AT, such as supervised intersection crossings, car-free zones and ‘Walking School Bus’ programs were recorded by principals. Safe routes to school relate to a combined engineering (e.g. speed humps, traffic crossings, speed limits, parking restrictions), education (e.g. Bike Education, Walk to School Programs), engagement (Local council, schools and community) and enforcement (law enforcers/police) approach which is promoted by Vicroads (Victorian Department of Transport) through their Safe Routes to School Approach [22]. Principals also recorded how adequate they perceived the indoor and outdoor play space to be at their school. The school’s physical environment was captured via the audit tool with questions around the availability of play equipment, sporting fields, gymnasiums and grassed areas for play as well as access to secure bike parking.

School enrolment numbers and socioeconomic position (based on the schools’ Index of Community Socio-Educational Advantage (ICSEA) scores) were obtained from the Australian Curriculum, Assessment and Reporting Authority website [23]. School ICSEA score is a measure of the school community’s socio-educational background, derived from reported parent/guardian occupation, parental/guardian income, geographic location and proportion of indigenous students [23].

#### Data management

Self-report measures of PA duration over the preceding 7 days were converted to a binary variable indicating adherence to the PA component of Australia’s 24-h movement guidelines of ≥60 min/day of moderate to vigorous physical activity (MVPA) [24]. Students indicated which transport mode they spent the most time doing during each trip to or from school and the predominant mode was used to classify active or non-active transport. Those reporting usually using AT (cycling, walking, or other active) either to or from school or in both directions over the preceding 7 days were classified as AT users.

For accelerometry, activity was recorded at a 30 Hz sample rate and analysed using a 5-s epoch with non-wear time calculated using the Toriano criteria of 60mins of consecutive zeroes with 1–2 min of tolerance of counts between 0 and 100 [25]. Wear time was considered valid if the device had been worn for ≥500 min/day over a minimum of 3 days. PA intensity was determined using metabolic equivalent units (METs) with moderate-to-vigorous PA defined as ≥4.0 METs using the Freedson age-specific counts per minute cut-points on the Vertical Axis (Axis 1) [26]. Average MVPA time per day, was calculated as total MVPA time divided by the number of valid wear days [27]. Students who achieved an average of ≥60mins/day MVPA were considered to have met the PA component of the 24-h movement guideline.

School ICSEA scores were dichotomised based on the national average score of 1000, to be classified as low (ICSEA≤999) or high (ICSEA≥1000) socioeconomic advantage [23]. Using section 43 [1] of the Australian Education Act 2013 [28] school enrolment was categorised into one of four size categories: Very small schools (< 15 students), small schools (15 to 200 students), medium Schools (201 to 299 students) and large schools (≥300 students). Schools that had measurement data (height and weight) for fewer than 10 students were excluded from this analysis (*n* = 22 schools).

**Fig. 1:**
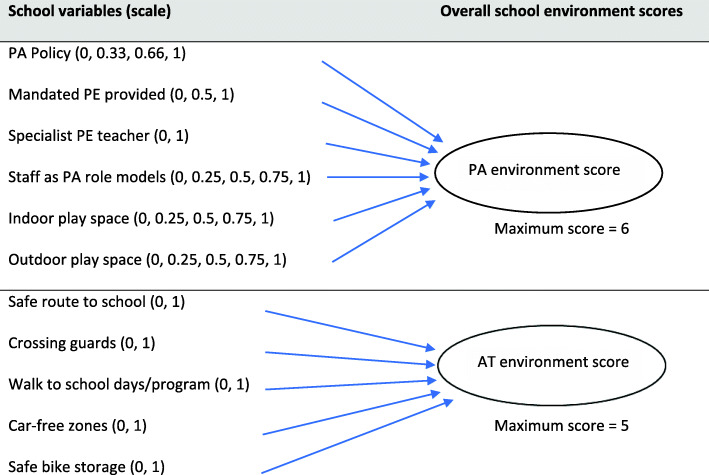
Composition of physical activity and active transport environment scores

School environment variables were either binary with no/yes responses (scored as 0/1) or answered on a Likert scale (see Supplementary File 2). PA and AT school environment scores were created by combining key variables (See Fig. 1) noted in the current literature as being associated with PA and AT behaviours [13, 15, 16, 29] and excluding those variables with low variability, such as access to grassed play area and access to bike storage (present in all participating schools). The total PA and AT environment scores were then recoded into tertiles and categorised as high, medium or low. A ‘high’ score indicated a high level of PA or AT promoting environment characteristics within the school.

### Statistical analysis

The proportion of students with overweight/obesity and of students meeting PA recommendations and using active transport was compared between genders using mixed effects logistic models, while the accelerometry outcomes were compared between genders using linear mixed models. Mixed-effects logistic models were fitted to estimate the association between the exposures, i.e. school PA or AT environment score (low, medium, high), and student outcomes, i.e. meeting PA recommendations, using AT and weight status (combined overweight/obesity). The same models were fitted adjusting for student gender, socio economic status (measured by school ICSEA) and school size (small, medium, large) [30]. School was included in all models as a random effect to account for clustering.

### Ethics

This project was approved by the Deakin University Human Research Ethics Committee (DUHREC2014–279), the Victorian Department of Education and Training (DET 2015_002622) and the Catholic Archdioceses of Sandhurst and Ballarat.

## Results

The surveillance systems achieved a school response rate of 65% and student response rate of 80%. The analysed sample included herein achieved 54/146 (37%) schools and 3360/5376 (64%) students after schools with < 10 measured students were removed (*n* = 22). In our sample, a large proportion (59.3%) of schools were ‘small’ (≥15 to < 200 students) and were from the government sector (79.6%) (Table 1). The majority of schools (59.3%) had an ICSEA score below the national average, indicating greater socioeconomic disadvantage. Further, almost two-thirds of the sample of students (62.6%) attended these schools with lower-socioeconomic position. Most schools reported a physical activity policy or practice (90.2%), a PE specialist teacher present (81.5%) and around two-thirds reported they provided the mandated amount of PE and SE time (67.3%). Supplementary Table 1 also summarises PA and AT policy characteristics by school sector, socioeconomic position and school size.

**Table 1:** School demographic and physical activity and active transport environment and policy characteristics

	Schools		Students	
	N	(%)	N	(%)
**Total Schools**	54	100	3360	100
**School size**				
Small (≥15 to 199 students)	32	59.3	1109	33.0
Medium (200 to 299 students)	15	27.8	1058	31.5
Large (≥ 300 students)	7	13.0	1193	35.5
**School sector**				
Government	43	79.6	2698	80.3
Catholic	7	13.0	505	15.0
Independent	4	7.4	157	4.7
**School socio-economic position**				
Low (ICSEA ≤999)	32	59.3	2103	62.6
High (ICSEA ≥1000)	22	40.7	1257	37.4
**School presence of healthy PA policy, PA and AT environment components**				
Physical activity policy or practice	46	90.2	3016	94.3
School provides equal to or more than mandated PE + SE time	35	67.3	2171	66.5
PE Specialist teacher available at school	44	81.5	3025	90.0
Teachers reported to be very good/good role models for PA	36	67.9	2480	75.0
Space for indoor play rated as good/very good	25	46.3	1875	55.8
Space for outdoor play rated as good/very good	53	98.2	3225	95.9
School promotes safe routes for walking and cycling to school	24	45.3	1843	57.2
School has a crossing guard at intersections around school environment	26	49.0	2200	68.2
School organises walking events (e.g. walk to school days)	42	79.3	2646	82.0
School has car-free zones	16	30.2	1083	33.6
School has secure bicycle racks	24	52.2	1876	65.5

*Abbreviations: PE* Physical Education, *SE* Sport Education, *PA* Physical activity, *AT* Active Transport, *ICSEA* Index of Community Socio-Educational Advantage

### Student level data

No gender differences were reported for students using active transport or for those classified as having overweight/obesity (Table 2). However, girls had significantly lower odds of reporting meeting PA recommendations than boys over 7- and ≥ 5–day criteria and similar gender differences were found in analysis of the accelerometry data.

**Table 2:** Self-reported meeting physical activity recommendations, using active transport to/from school and measured accelerometry and overweight/obese by gender

	Mean/Prop				Odds ratio (95%CI)	
	Boys	Girls	p	Boys	Girls	p
**Self-report**				(*n* = 1150)	(*n* = 1090)	
Meeting PA guidelines past 7-day self-report^a^ (%)	22.9	14.0	*p* < 0.001	Reference	0.54 (0.42, 0.67)	*p* < 0.001
Meeting PA guidelines ≥5-day self-report^a^ (%)	43.0	32.1	*p* < 0.001	Reference	0.60 (0.50, 0.71)	*p* < 0.001
Using Active Transport to and/or from school (%)	34.6	34.2	NS	Reference	0.94 (0.78, 1.14)	NS
Overweight and Obesity^b^ (%)	33.2	33.8	NS	Reference	1.03 (0.89, 1.20)	NS
**Accelerometry**				(*n* = 453)	(*n* = 446)	
Valid wear (days) (Mean + SD)	5.1 (1.7)	5.3 (1.6)	NS	Reference	0.24 (0.0, 0.48)	*P* = 0.05
Daily wear-time (min/day) (Mean + SD)	737.8 (121.9)	739.3 (122.8)	NS	Reference	1.41 (−16.6, 19.4)	NS
Daily Light PA (min/day) (Mean + SD)	157.2 (29.2)	155.3 (29.4)	NS	Reference	27.2 (−12.8, 67.2)	NS
Daily MVPA (min/day) (Mean + SD)	81.4 (24.2)	67.8 (20.6)	*p* < 0.001	Reference	−53.9 (−78.0, −29.7)	*p* < 0.001
Meeting PA guidelines^a^ (%)	80.6	62.0	*p* < 0.001	Reference	−0.97 (−1.32, −0.61)	*p* < 0.001

Notes: ^a^ Odds of meeting ≥ 60mins of Moderate-to-Vigorous Physical Activity/day, ^b^ WHO Cut-points (Overweight/Obesity) Odds of healthy BMI compared to overweight/obesity, *NS* not significant

#### Physical activity environments, active transport environments and student behaviours and weight status

The unadjusted and adjusted models (Table 3) showed no associations between school PA environment score or AT environment score and odds of being healthy weight compared to having overweight/obesity. There were no associations between the school PA environment scores and meeting PA recommendations either self-reported or measured using accelerometry Although, a gender-specific analysis (Supplementary Table 3) found girls were significantly more likely to meet the PA guidelines (measured using accelerometry) if they attended a school with a medium PAES score (OR 2.30, 95% CI 1.27, 4.16), when compared to girls attending low scoring schools. After adjustment for potential confounders, a higher AT environment score increased the odds of students reporting using AT to and/or from school when compared to the low ATES scoring schools (reference) with higher odds for medium (OR 3.15, 95%CI 1.67,5.94) and high (OR 3.71, 95%CI 1.80, 7.64) scoring schools, (*P* < 0.001). These findings were also reflected in the gender-specific models (Supplementary Table 2&3).

**Table 3:** Associations between students’ self- reported and objective physical activity, active transport use and weight status and both the physical activity and active transport environment scores

Overall	Odds ratio (95% confidence interval)						
**Unadjusted model(a) (overall** ***p*-value)**	*p* = 0.20	*p* = 0.25	*p* = 0.67	*p* = 0.37	**Unadjusted model(a) (overall** ***p*-value)**	*p* < 0.01	*p* = 0.66
**Physical activity environment score**	**7-day self-report PA** ^**1**^	**≥5-day self-report PA** ^**2**^	**Accelerometer PA** ^**3**^	**Weight status** ^**4**^	**Active transport environment score**	**Active transport to and from school**	**Weight status** ^**4**^
Low (reference)	1.0	1.0	1.0	1.0	**Low (reference)**	1.0	1.0
Medium	0.80 (0.58,1.09)	0.90 (0.65,1.25)	1.26 (0.74,2.13)	1.0 (0.81,1.23)	**Medium**	3.22 (1.72,6.03)*	0.92 (0.75,1.14)
High	0.76 (0.54,1.07)	0.74 (0.52,1.05)	1.01 (0.57,1.79)	0.87 (0.69,1.08)	**High**	3.55 (1.75,7.21)*	0.91 (0.74, 1.13)
**Adjusted model(b) (overall p-value)**	*p* < 0.01	*p* < 0.01	*p* < 0.01	*p* < 0.05	**Adjusted model(b) (overall p-value)**	*p* < 0.01	*p* < 0.05
**Physical activity environment score**	**7-day self-report PA** ^**1**^	**≥5-day self-report PA** ^**2**^	**Accelerometer PA** ^**3**^	**Weight status** ^**4**^	**Active transport environment score**	**Active transport to and from school**	**Weight status** ^**4**^
Low (reference)	1.00	1.00	1.00	1.00	**Low (reference)**	1.00	1.00
Medium	0.75 (0.52, 1.09)	0.80 (0.56, 1.15)	1.39 (0.78, 2.45)	1.09 (0.88, 1.35)	**Medium**	3.15 (1.67, 5.94)*	0.98 (0.81, 1.18)
High	0.74 (0.51, 1.07)	0.70 (0.49, 1.01)	1.0 (0.55, 1.82)	0.95 (0.77, 1.17)	**High**	3.71 (1.80, 7.64)*	0.97 (0.79, 1.19)

Notes: Model (a) Mixed logistic regression. Model (b) Mixed logistic regression adjusted for ICSEA, school size and sex and wear time for accelerometer measure physical activity. All models included school as a random effect. * *P* ≤ 0.05. (1) Meeting the physical activity guidelines on 7 days, (2) Meeting the physical activity guidelines on ≥5 days, (3) ≥60mins of MVPA/day on average, (4) WHO Cut-points, Odds of healthy BMI compared to overweight/obesity. Physical activity environment score, low score (5–7) medium (8) and high (9–11). Active transport environment score, low score (0–2) medium (3) and high (4, 5)

**Fig. 2:**
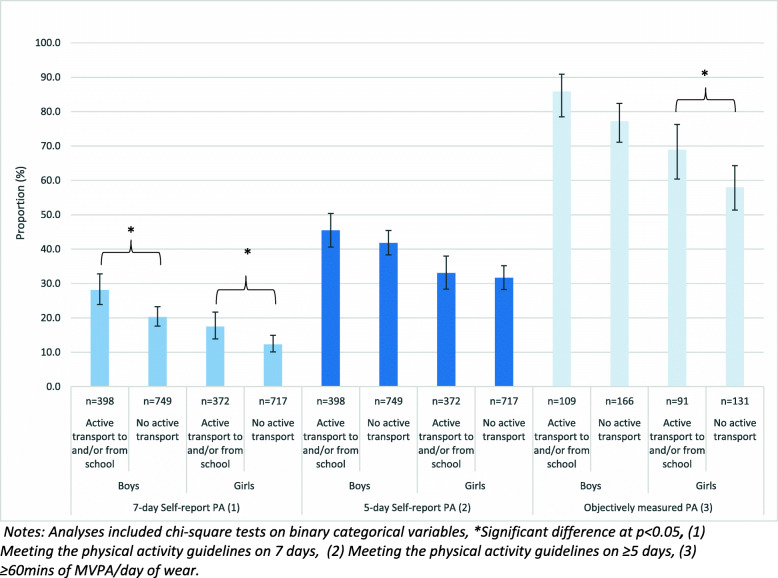
Proportion boys and girls meeting the physical activity guidelines by self-reported use of active transport to and/or from school. Notes: Analyses included chi-square tests on binary categorical variables, *Significant difference at *p* < 0.05, (1) Meeting the physical activity guidelines on 7 days, (2) Meeting the physical activity guidelines on ≥5 days, (3) ≥60mins of MVPA/day of wear

Figure 2 highlights that both boys (OR 1.59, 95%CI 1.19, 2.13) and girls (OR 1.56, 95%CI 1.08, 2.27) who reported using AT to and/or from school were also significantly more likely to meet 7-day self-report physical activity guidelines than those who did not report using AT. Additionally, girls who used AT to and/or from school were also significantly more likely do ≥60 min of MVPA/day of wear as recorded via accelerometry (OR 1.81, 95%CI 1.10, 2.97) (Table 4).

**Table 4:** Adjusted associations between self-reported use of active transport to and/or from school and students meeting the physical activity guidelines

		Odds ratio (95% confidence interval)		
Gender	Active Transport use	7-day self-report PA^**a**^	≥5-day self-report PA^**b**^	Accelerometer PA^**c**^
**Boys**	**No Active transport to school (reference)**	1.00	1.00	1.00
	**Active transport to and/or from school**	1.59 (1.19, 2.13)*	1.20 (0.92, 1.55)	1.81 (0.95, 3.43)
**Girls**	**No Active transport to school (reference)**	1.00	1.00	1.00
	**Active transport to and/or from school**	1.56 (1.08, 2.27)*	1.04 (0.78, 1.39)	1.81 (1.10, 2.97)*

Notes: Model Mixed logistic regression adjusted for ICSEA, school size and wear time for accelerometer measure physical activity. All models included school as a random effect. * *P* ≤ 0.05 ^a^ Meeting the physical activity guidelines on 7 days, ^b^ Meeting the physical activity guidelines on ≥ 5 days, ^c^ ≥60mins of MVPA/day on average, * Significant difference at *p* < 0.05. Adjusted for ICSEA, school size and wear time for accelerometer measure physical activity. Models included school as a random effect

## Discussion

This study explored the associations between policy, practice, structural and cultural elements within the school environment and three key health outcomes/behaviours; proportions of students meeting PA recommendations, using active transport (AT) to and from school, and being classified as having overweight or obesity. We found no association between the school’s physical activity environment and odds of meeting PA recommendations overall, or the students’ odds of being a healthy weight compared to having overweight or obesity. In stratified analysis, an association was found between physical activity environment and objectively measured PA guideline adherence for girls only. An association was observed between increased quality of the AT environment and AT participation. Further, those students who used active transport to and/or from school were more likely to meet PA recommendations than their counterparts who did not use active transport. This demonstrates the important role schools can play in encouraging active transport to and from school to support children in meeting physical activity recommendations.

We also found low levels of physical activity among primary school children, though boys were more active than girls, in both self-report and objective data. Both the low levels of PA and the gendered differences in PA were consistent with existing literature across all ages [4, 31]. The International Children’s Accelerometry Database (ICAD), that consists of ActiGraph accelerometer data from 20 studies in ten countries, has demonstrated that boys were more active than girls, and that physical activity levels were not associated with the students’ weight status [32]. Importantly, our study found that attending a school with a higher PA environment score was associated with higher PA guidance adherence among girls but not boys. Highlighting an important area for further intervention research. To the best of our knowledge, this is the first study in Australia to comprehensively examine policies and practices around school PA and AT. We found that approximately two-thirds of schools provided at least the minimum mandated amount of PE and SE, with Government schools less likely than Independent schools to provide the mandated amount. Providing the mandated amount of PE and SE, or greater, also differed by school socio-educational position (ICSEA) with fewer schools that were classified as low ICSEA (more disadvantaged) meeting the mandate than schools classified as high ICSEA.

A recent review showed that PE and sport in schools can contribute to increased levels of physical activity in students and that school-based programs also contribute to physical activity levels later in life [33]. The same review cautions that mere provision of PE and sport is not sufficient and that the amount, and particularly the quality, of programs provided is crucial. It also notes the benefits of PE and sport are realised when programs are engaging, diverse and enjoyable and managed by committed and trained specialist teachers [33]. Overall, our study found the reported rate of schools having a specialist PE teacher on staff was high and differed little by school sector and ICSEA classification. A higher proportion of schools classified as large (by enrolments) reported having a specialist PE teacher compared to small and medium sized schools. However, the proportion of small and medium sized schools having a specialist PE teacher was still high. This is encouraging given that a quasi-experimental study showed that having a specialist PE teacher deliver PE resulted in significantly increased levels of physical fitness, explosive strength, running speed and flexibility in students when compared to PE delivered by generalist teachers [34].

Our finding of no association between quality of school physical activity environments and student weight status is consistent with Haddad et al’s (2018) study of approximately 2500 school children in the same age group set in both rural (32%) and metropolitan (68%) areas of South Australia [35]. Haddad and colleagues found the quality of the home environment was more strongly associated with students’ measured BMI than the school environment. Contrary to our findings, the large cross-sectional multi-country ISCOLE study involving 6797 school children (aged 9-11 years) found that children who reported using active transport were less likely to be obese (odds ratio = 0.72, 95% CI 0.60–0.87) and had a lower BMI z-scores than those who did not report active transport [36]. Our results are also consistent with the findings of a previous systematic review of 19 studies that showed providing environments that support AT increased AT participation [37]. Similarly, our findings suggest that providing supportive school policies and infrastructure for active transport can increase AT [38]. This builds on previous studies including one of more than 1000 children in Oregon, United States, which demonstrated that more supportive and safe active transport environments around schools increased the likelihood that children would participate in active transport to and from school [15] .

Principals reported the promotion of walk or ride to schools days was generally high across all school sizes, sectors and ICSEA classifications and these have been shown to be effective in increasing students’ active transport to school [39]. Our results showed that the provision of crossing guards at intersections around the school varied greatly between school sector, school size and ICSEA classification but was generally reported to be quite low, with the exception of large sized schools. The reported provision of secure bike racks also varied but was lower in Government schools, schools classified as high ICSEA and small schools. The reported promotion of safe routes for walking and cycling to school was low in Government, low ICSEA classified and small sized schools. This is despite research conducted in 577 US schools showing that schools have approximately three times the odds of having ≥26% of students using active modes of transport to school when they provide crossing guards (OR 3.3, 95%CI 2.9, 6.0, *p* < 0.001), bike racks (OR 2.7, 95%CI 1.2, 5.8, *p* = 0.01) or promotional material around walking and cycling to school (OR 2.9, 95%CI 1.7, 5.1, *p* < 0.001) [40].

### Strengths

Data used in this study were collected across two regional areas in Victoria using opt-out recruitment and high student (80%) and school (65%) response rates providing a representative study of the schools and students in these regions. Previous studies used an opt-out versus and opt-in recruitment method provides more accurate estimations of childhood BMI-z and weight status outcomes [41] and a range of differences across self-report behavioural outcomes as well [42]. This study also utilized both subjective self-report and objective accelerometer data to determine the number of children meeting the PA recommendations. Dollman et al. [43] note that objective measures of PA, whilst not perfect at measuring all activity, such as cycling or swimming, do address the key limitations of subjective measures of PA, particularly memory limitations in young people. The inclusion of accelerometry strengthens our analysis as it is well-known that self-reported physical activity data, particularly in children, often suffers from recall and social desirability bias [44]. Additionally, whilst accelerometers provide accurate estimates of physical activity energy expenditure, they provide no information on the domains in which they occur (e.g. household, occupation, leisure-time, transport, PE) [43]. Therefore, self-report measures are complimentary to examine specific activity domains and examine the effectiveness of specific policies/practices to improve PA.

### Limitations

The cross-sectional nature of this study limits our ability to make inference about causality between the school environment and PA and AT levels. It may be that the school environment and policies have been improved in response to school community concerns about students’ physical activity and active transport behaviours, which we would not be able to identify in the current study. With the exception of weight status and accelerometry, all student outcomes and principals’ environment audits were collected using self-report surveys and therefore subject to recall/social desirability bias associated with self-report data. The school environment audits were self-reported by school principals, and as noted by Turner et al. [45] there is a need for more objective measures of school environments to enable better understand on the associations between environment and weight status in children.

### Future research

While we examined the cross-sectional associations between environments, PA, AT and weight status future longitudinal research is needed to understand the causal relationships. A second area for future research would be to develop a more nuanced understanding of the features and quality of the environment that are most strongly associated with subsequent PA behaviours. A comprehensive approach would comprise collation of large data sets alongside qualitative studies and modelling studies. Future research could gain more detail on the actual distance travelled to or from school and similarly the quality of environments, the influence of role modelling and other prompts (e.g. signage, placement in curriculum). Additionally, future research should examine whether these relationships vary by key characteristics such as school type (e.g. government, independent or catholic), remoteness (e.g. inner regional, outer regional or remote) and socioeconomic position.

### Implications for practice

Whilst it is well known that adherence to physical activity recommendations in Australia is low [1], efforts to change this through interventions at both and individual and environmental levels have had variable impacts, particularly when scaled-up or implemented in real-world settings [46]. Schools are a key environment in which to address childhood rates of inactivity through interventions focused on the physical, policy, practice and curriculum environment. Whilst our cross-sectional data showed a significant association between the number of participants meeting the physical activity recommendations and the PAES only for girls, the latest Cochrane review noted that interventions in the school environment have significantly increased levels of MVPA [47]. However other reviews warned that the small effect size and moderate risk of bias in these types of interventions means more research is needed to understand how interventions targeting physical activity environments and to understand both immediate and long term effects on children’s PA [48].

## Conclusion

This cross-sectional study provides evidence that school physical activity policies and practices in Victorian schools were not associated with measured weight status, and were associated with adherence to PA recommendations only among girls. School active transport policies and practices were strongly associated with students’ active transport behaviours. This is of particular note as those who used active transport to and/or from school were more likely to meet physical activity recommendations. Improvements in active transport policies and practices in schools provides a potential pathway for increasing the proportion of students meeting the physical activity recommendations and promoting improved health and wellbeing outcomes.

## Supplementary Information

**Additional file 1: Supplementary Table 1.** School level environment characteristics by school sector, socioeconomic position (ICSEA) and school enrolment size. **Supplementary Table 2.** Associations between students’ self- reported and objective physical activity, active transport use and weight status and both the physical activity and active transport environment scores among boys. **Supplementary Table 3.** Associations between students’ self- reported and objective physical activity, active transport use and weight status and both the physical activity and active transport environment scores among girls.

**Additional file 2.** Primary School Questionnaire.

**Additional file 3.** Primary School – Environmental Audit.

## Data Availability

The data are held within Deakin University and due to ethical constraints cannot be shared.

## Abbreviations

PA: Physical activity
PE: Physical Education
AT: Active Transport
MVPA: Moderate-to-vigorous physical activity
ATES: Active Transport Environment Score
PAES: Physical Activity Environment Score
ICSEA: Index of Community Socio-Educational Advantage
SE: Sport Education
BMI: Body Mass Index
OR: Odds Ratio
