# Supplementary material for: Association between the school physical activity environment, measured and self-reported student physical activity and active transport behaviours in Victoria, Australia
Source: Int J Behav Nutr Phys Act. 2021 Jun 22;18:79. doi: 10.1186/s12966-021-01151-6 (PMC8220765; doi:10.1186/s12966-021-01151-6)
Supplement: Supplementary file 1 — Additional file 1: Supplementary Table 1. School level environment characteristics by school sector, socioeconomic position (ICSEA) and school enrolment size. Supplementary Table 2. Associations between students’ self- reported and objective physical activity, active transport use and weight status and both the physical activity and active transport environment scores among boys. Supplementary Table 3. Associations between students’ self- reported and objective physical activity, active transport use and weight status and both the physical activity and active transport environment scores among girls. [file 12966_2021_1151_MOESM1_ESM.docx]

***Supplementary Table 1. School level environment characteristics by school sector, socioeconomic position (ICSEA) and school enrolment size***

| **Reported presence of healthy policy and physical activity environment components** | **Sector** | | **ICSEA** | | **School Size** | | |
| --- | --- | --- | --- | --- | --- | --- | --- |
|  | Government  (N = 43) | Catholic & Independent (N = 11) | Low  (N = 32) | High  (N = 22) | Small  (N = 32) | Medium  (N = 15) | Large  (N = 7) |
| **Physical Activity policy, practice & environment** |  |  |  |  |  |  |  |
| Physical activity policy (written or under development) + Practice (%) | 88.4 | 100.0 | 90.6 | 89.5 | 90.3 | 84.6 | 100.0 |
| School provides equal to or more than mandated PE + SE time (%) | 65.9 | 81.8 | 60.0 | 77.3 | 66.7 | 66.7 | 71.4 |
| PE Specialist teacher available at school (%) | 81.4 | 81.8 | 84.4 | 77.3 | 78.1 | 80.0 | 100.0 |
| Teachers reported to be very good/good role models for PA (%) | 67.4 | 70.0 | 65.6 | 71.4 | 62.5 | 85.7 | 57.1 |
| Space for indoor play rated as good/very good (%) | 46.5 | 45.5 | 46.9 | 45.5 | 46.9 | 33.3 | 71.4 |
| Space for outdoor play rated as good/very good (%) | 97.7 | 100.0 | 100.0 | 95.5 | 100.0 | 100.0 | 85.7 |
| **PA Environment Score - Mean (SD)** | 4.1 (0.7) | 4.1 (0.7) | 4.1 (0.7) | 4.0 (0.7) | 4.1 (0.8) | 3.9 (0.6) | 4.5 (0.3) |
| **Active Transport policy, practice & environment** |  |  |  |  |  |  |  |
| School promotes safe routes for walking and cycling to school (%) | 40.5 | 63.6 | 31.3 | 66.7 | 37.5 | 53.3 | 66.7 |
| School has a crossing guard at intersections around school environment (%) | 52.4 | 36.4 | 59.4 | 33.3 | 37.5 | 53.3 | 100.0 |
| School organises walking events (e.g. walk to school days) (%) | 76.2 | 90.9 | 78.1 | 81.0 | 81.3 | 73.3 | 83.3 |
| School has car-free zones (%) | 26.2 | 45.5 | 31.3 | 28.6 | 28.1 | 26.7 | 50.0 |
| School has secure bicycle racks (%) | 47.2 | 70.0 | 55.2 | 47.1 | 44.4 | 61.5 | 66.7 |
| **AT Environment Score - Mean (SD)** | 2.3 (1.3) | 3.0 (1.1) | 2.5 (1.2) | 2.4 (1.4) | 2.2 (1.3) | 2.6 (0.9) | 3.1 (1.6) |

*Abbreviations: PE = Physical Education; SE = Sport Education, PA = Physical activity, ICSEA = Index of Community Socio-Educational Advantage.*

***Supplementary Table 2. Associations between students’ self- reported and objective physical activity, active transport use and weight status and both the physical activity and active transport environment scores among boys***

| **Boys** | **Odds ratio (95% confidence interval)** | |  |  |  |  |  |
| --- | --- | --- | --- | --- | --- | --- | --- |
| **Unadjusted model(a) (overall p-value)** | p=0.24 | p=0.43 | p=0.52 | p=0.11 | **Unadjusted model(a) (overall p-value)** | p<0.01 | p=0.06 |
| **Physical activity environment score** | **7-day self-report PA ^1^** | **≥5-day self-report PA ^2^** | **Accelerometer PA ^3^** | **Weight status ^4^** | **Active transport environment score** | **Active transport to and from school** | **Weight status ^4^** |
| Low (reference) | 1.0 | 1.0 | 1.0 | 1.0 | **Low (reference)** | 1.0 | 1.0 |
| Medium | 0.76 (0.51,1.14) | 0.82 (0.55,1.23) | 0.61 (0.26,1.44) | 0.86 (0.65,1.14) | **Medium** | 3.41 (1.76,6.63)* | 0.75 (0.58,0.96)* |
| High | 0.71 (0.46,1.11) | 0.77 (0.50,1.18) | 0.80 (0.32,2.01) | 0.74 (0.56,0.98) | **High** | 3.77 (1.80,7.87)* | 0.79 (0.60, 1.02) |
| **Adjusted model(b) (overall p-value)** | P=0.18 | p=0.20 | p=0.56 | p<0.05 | **Adjusted model(b) (overall p-value)** | p<0.01 | p<0.05 |
| **Physical activity environment score** | **7-day self-report PA ^1^** | **≥5-day self-report PA ^2^** | **Accelerometer PA ^3^** | **Weight status ^4^** | **Active transport environment score** | **Active transport to and from school** | **Weight status ^4^** |
| Low (reference) | 1.00 | 1.00 | 1.00 | 1.00 | **Low (reference)** | 1.00 | 1.00 |
| Medium | 0.75 (0.48, 1.18) | 0.79 (0.51, 1.20) | 0.59 (0.23, 1.48) | 0.95 (0.70, 1.29) | **Medium** | 3.27 (1.65, 6.47)* | 0.84 (0.64, 1.10) |
| High | 0.75 (0.47, 1.17) | 0.79 (0.52, 1.21) | 0.76 (0.29, 2.02) | 0.81 (0.60, 1.09) | **High** | 3.80 (1.78, 8.13)* | 0.86 (0.65, 1.15) |

Notes: Model (a) Mixed logistic regression. Model (b) Mixed logistic regression adjusted for ICSEA, school size and wear time for accelerometer measure physical activity. All models included school as a random effect. * P≤ 0.05. (1) Meeting the physical activity guidelines on 7 days, (2) Meeting the physical activity guidelines on ≥5 days, (3) ≥60mins of MVPA/day of wear, (4) WHO Cut-points, Odds of healthy BMI compared to overweight/obesity. Physical activity environment score, low score (5-7) medium (8) and high (9-11). Active transport environment score, low score (0-2) medium (3) and high (4-5).

***Supplementary Table 3. Associations between students’ self- reported and objective physical activity, active transport use and weight status and both the physical activity and active transport environment scores among girls***

| **Girls** | **Odds ratio (95% confidence interval)** | |  |  |  |  |  |
| --- | --- | --- | --- | --- | --- | --- | --- |
| **Unadjusted model(a) (overall p-value)** | p=0.79 | p=0.36 | p=0.05* | p=0.76 | **Unadjusted model(a) (overall p-value)** | p<0.01 | p=0.91 |
| **Physical activity environment score** | **7-day self-report PA ^1^** | **≥5-day self-report PA ^2^** | **Accelerometer PA ^3^** | **Weight status ^4^** | **Active transport environment score** | **Active transport to and from school** | **Weight status ^4^** |
| Low (reference) | 1.0 | 1.0 | 1.0 | 1.0 | **Low (reference)** | 1.0 | 1.0 |
| Medium | 0.87 (0.51,1.47) | 1.02 (0.65,1.61) | 2.03 (1.14,3.64)* | 1.10 (0.85,1.41) | **Medium** | 3.17 (1.71,5.87)* | 1.06 (0.82,1.36) |
| High | 0.84 (0.47,1.49) | 0.72 (0.43,1.20) | 1.18 (0.64,2.19) | 1.04 (0.79,1.36) | **High** | 3.42 (1.74,6.73)* | 1.03 (0.80, 1.34) |
| **Adjusted model(b) (overall p-value)** | p=0.85 | p=0.36 | p=0.09 | p=0.14 | **Adjusted model(b) (overall p-value)** | p<0.01 | p=0.22 |
| **Physical activity environment score** | **7-day self-report PA ^1^** | **≥5-day self-report PA ^2^** | **Accelerometer PA ^3^** | **Weight status ^4^** | **Active transport environment score** | **Active transport to and from school** | **Weight status ^4^** |
| Low (reference) | 1.00 | 1.00 | 1.00 | 1.00 | **Low (reference)** | 1.00 | 1.00 |
| Medium | 0.75 (0.40, 1.39) | 0.81 (0.49, 1.34) | 2.30 (1.27, 4.16)* | 1.26 (0.93, 1.69) | **Medium** | 3.16 (1.87, 5.92)* | 1.14 (0.87, 1.50) |
| High | 0.74 (0.40, 1.37) | 0.63 (0.37, 1.06) | 1.25(0.68, 2.33) | 1.11 (0.82, 1.49) | **High** | 3.69 (1.84, 7.40)* | 1.10 (0.83, 1.46) |

Notes: Model (a) Mixed logistic regression. Model (b) Mixed logistic regression adjusted for ICSEA, school size and wear time for accelerometer measure physical activity. All models included school as a random effect. * P≤ 0.05. (1) Meeting the physical activity guidelines on 7 days, (2) Meeting the physical activity guidelines on ≥5 days, (3) ≥60mins of MVPA/day of wear, (4) WHO Cut-points, Odds of healthy BMI compared to overweight/obesity. Physical activity environment score, low score (5-7) medium (8) and high (9-11). Active transport environment score, low score (0-2) medium (3) and high (4-5).
