# Supplementary material for: Association between the school physical activity environment, measured and self-reported student physical activity and active transport behaviours in Victoria, Australia
Source: Int J Behav Nutr Phys Act. 2021 Jun 22;18:79. doi: 10.1186/s12966-021-01151-6 (PMC8220765; doi:10.1186/s12966-021-01151-6)
Supplement: Supplementary file 2 — Additional file 2. Primary School Questionnaire. [file 12966_2021_1151_MOESM2_ESM.doc]

Primary School Questionnaire

(Demographic, Physical activity and Active Transport components)

| Grade 4 & 6 Student Questionnaire |
| --- |

**First Name Last Name**

|  |  |  |  |  |  |  |  |  |  |  |  |  |  |  |  |  |  |  |  |  |  |  |  |  |  |
| --- | --- | --- | --- | --- | --- | --- | --- | --- | --- | --- | --- | --- | --- | --- | --- | --- | --- | --- | --- | --- | --- | --- | --- | --- | --- |

**Date of Birth (Birthday) School Name**

| **D** | **D** | **/** | **M** | **M** | **/** | **Y** | **Y** |  |  |  |  |  |  |  |  |  |  |  |  |  |  |  |  |  |
| --- | --- | --- | --- | --- | --- | --- | --- | --- | --- | --- | --- | --- | --- | --- | --- | --- | --- | --- | --- | --- | --- | --- | --- | --- |

**1. Which town/city/suburb do you usually live in? 2. What is the postcode?**

|  |  |  |  |  |  |  |  |  |  |  |  |  |  |  |  |  |  |  |  |  |  |
| --- | --- | --- | --- | --- | --- | --- | --- | --- | --- | --- | --- | --- | --- | --- | --- | --- | --- | --- | --- | --- | --- |

| **3. Are you a boy or girl?**  ⃝ Boy  ⃝ Girl | **4. Are you an Aboriginal or Torres Strait Islander?**  ⃝ No  ⃝ Yes, Aboriginal  ⃝ Yes, Torres Strait Islander  ⃝ Yes, Both Aboriginal and Torres Strait Islander  ⃝ Don’t Know |
| --- | --- |
| **5. Do you speak a language other than English at home?**  ⃝ No, English only  ⃝ Yes, Italian  ⃝ Yes, Greek  ⃝ Yes, Cantonese  ⃝ Yes, Arabic  ⃝ Yes, Mandarin  ⃝ Yes, Vietnamese  ⃝ Yes, other – please specify   |  |  |  |  |  |  |  |  |  |  |  |  |  | | --- | --- | --- | --- | --- | --- | --- | --- | --- | --- | --- | --- | --- | | **6. In which country were you born?**  ⃝ Australia  ⃝ England  ⃝ New Zealand  ⃝ Italy  ⃝ Vietnam  ⃝ India  ⃝ Scotland  ⃝ Other- please specify   |  |  |  |  |  |  |  |  |  |  |  |  |  | | --- | --- | --- | --- | --- | --- | --- | --- | --- | --- | --- | --- | --- | |
| **7. What is your ancestry?** (***select up to two***)  ⃝ English ⃝ German  ⃝ Irish ⃝ Chinese  ⃝ Scottish ⃝ Australian  ⃝ Italian ⃝ Other – please specify | |  |  |  |  |  |  |  |  |  |  |  |  |  | | --- | --- | --- | --- | --- | --- | --- | --- | --- | --- | --- | --- | --- | |

**8. Mark how many minutes of physical activity you did on each of the past 7 days. Include physical activity during physical education class, lunch, after school, evenings, and spare time. Physical activities include skating, bike riding, running, skateboarding/rollerblading and any other physical activities that make you sweat, breathe harder or be “out of breath”.**

|  | **None** | **1 to 14 minutes** | **15 to 29 minutes** | **30 to 59 minutes** | **1 to 2 hours** | **More than 2 hours** |
| --- | --- | --- | --- | --- | --- | --- |
| **Monday** | ⃝ | ⃝ | ⃝ | ⃝ | ⃝ | ⃝ |
| **Tuesday** | ⃝ | ⃝ | ⃝ | ⃝ | ⃝ | ⃝ |
| **Wednesday** | ⃝ | ⃝ | ⃝ | ⃝ | ⃝ | ⃝ |
| **Thursday** | ⃝ | ⃝ | ⃝ | ⃝ | ⃝ | ⃝ |
| **Friday** | ⃝ | ⃝ | ⃝ | ⃝ | ⃝ | ⃝ |
| **Saturday** | ⃝ | ⃝ | ⃝ | ⃝ | ⃝ | ⃝ |
| **Sunday** | ⃝ | ⃝ | ⃝ | ⃝ | ⃝ | ⃝ |

**9. For each of the past 7 days, mark how many hours (outside of school) did you spent sitting or lying down looking at a screen. Think about the time you spent watching TV and movies, playing video games, video chatting, text messaging, or surfing internet sites like Twitter or YouTube, for example.**

|  | **None** | **Less than 1 hour a day** | **1 to 2 hours a day** | **More than 2 hours but less than 5 hours a day** | **5 or more hours a day** |
| --- | --- | --- | --- | --- | --- |
| **Monday** | ⃝ | ⃝ | ⃝ | ⃝ | ⃝ |
| **Tuesday** | ⃝ | ⃝ | ⃝ | ⃝ | ⃝ |
| **Wednesday** | ⃝ | ⃝ | ⃝ | ⃝ | ⃝ |
| **Thursday** | ⃝ | ⃝ | ⃝ | ⃝ | ⃝ |
| **Friday** | ⃝ | ⃝ | ⃝ | ⃝ | ⃝ |
| **Saturday** | ⃝ | ⃝ | ⃝ | ⃝ | ⃝ |
| **Sunday** | ⃝ | ⃝ | ⃝ | ⃝ | ⃝ |

**10. Do you have a TV or electronic device with a screen in your bedroom?**

⃝ Yes

⃝ No

***11.*** ***During the past 7 days, how did you usually get to and from school? (If you use two or more modes of travel, choose the one that you spend most time doing)***

|  | **To School** |  | **From school** |
| --- | --- | --- | --- |
| ⃝ | Car | ⃝ | Car |
| ⃝ | School bus | ⃝ | School bus |
| ⃝ | Public bus, Train or Tram | ⃝ | Public bus, Train or Tram |
| ⃝ | Walking | ⃝ | Walking |
| ⃝ | Cycling | ⃝ | Cycling |
| ⃝ | Other active | ⃝ | Other active |
| ⃝ | Other inactive | ⃝ | Other inactive |

**12. Did your activities over the past 7 days represent a typical week for you?**

⃝ Yes

⃝ No

| **13. Do you consider yourself:**  ⃝ Very overweight  ⃝ Slightly overweight  ⃝ About the right weight  ⃝ Slightly underweight  ⃝ Very underweight | **14. In general, compared to other people your age, how would you rate your athletic ability?**  ⃝ Excellent  ⃝ Good  ⃝ Fair  ⃝ Poor |
| --- | --- |
| **15. How physically active do you consider your father (or male caregiver) to be?**  **⃝ Active**  **⃝ Somewhat active**  **⃝ Inactive**  **⃝ I don’t know** | **16. How physically active do you consider your mother (or female caregiver) to be?**  **⃝ Active**  **⃝ Somewhat active**  **⃝ Inactive**  **⃝ I don’t know** |
| **17. How much do your parent(s) or guardian(s) support you to be physically active?** (e.g. driving you to team games, buying you sporting equipment, etc.)  ⃝ Very supportive  ⃝ Supportive  ⃝ Unsupportive  ⃝ Very unsupportive | **18. How much do your parent(s) or guardian(s) encourage you to be physically active?**  ⃝ Strongly encourage  ⃝ Encourage  ⃝ Do not encourage or discourage  ⃝ Discourage  ⃝ Strongly discourage |

Phyical activity and Active Transport question item sources include:

*Card A, Manske S, Mammen G, King M, Gleddie D, Schwartz M. Core Indicators and Measures of Youth Health Physical Activity & Sedentary Behaviour Module: Indicators and Questions to use with Youth Respondents and/or School Setting Assessment. Newfoundland, Canada: Memorial University of Newfoundland; 2012.*

*Wong SL, Leatherdale ST, Manske SR. Reliability and validity of a school-based physical activity questionnaire. Med Sci Sports Exerc. 2006;38(9):1593-600.*
